# Biomarkers and phenotypic expression in Alzheimer’s disease: exploring the contribution of frailty in the Alzheimer’s Disease Neuroimaging Initiative

**DOI:** 10.1007/s11357-020-00293-y

**Published:** 2020-11-19

**Authors:** Marco Canevelli, Ivan Arisi, Ilaria Bacigalupo, Andrea Arighi, Daniela Galimberti, Nicola Vanacore, Mara D’Onofrio, Matteo Cesari, Giuseppe Bruno, for the Alzheimer’s Disease Neuroimaging Initiative

**Affiliations:** 1 Department of Human Neuroscience, “Sapienza” University of Rome, Rome, Italy; 2 National Center for Disease Prevention and Health Promotion, Italian National Institute of Health, Rome, Italy; 3 Bioinformatics Facility, European Brain Research Institute (EBRI) Rita Levi-Montalcini, Rome, Italy; 4 Institute of Translational Pharmacology (IFT), CNR, Rome, Italy; 5 Neurodegenerative Diseases Unit, Fondazione IRCCS Ca’ Granda Ospedale Maggiore Policlinico, Milan, Italy; 6 Dino Ferrari Center, University of Milan, Milan, Italy; 7 Department of Biomedical, Surgical and Dental Sciences, University of Milan, Milan, Italy; 8 Genomics Facility, European Brain Research Institute (EBRI) Rita Levi-Montalcini, Rome, Italy; 9 Department of Clinical Sciences and Community Health, University of Milan, Milan, Italy; 10 Geriatric Unit, IRCCS Istituti Clinici Scientifici Maugeri, Milan, Italy

**Keywords:** Alzheimer’s disease, Frailty, Biomarkers, Aging, Dementia

## Abstract

The present study aimed at investigating if the main biomarkers of Alzheimer’s disease (AD) neuropathology and their association with cognitive disturbances and dementia are modified by the individual’s frailty status. We performed a cross-sectional analysis of data from participants with normal cognition, mild cognitive impairment (MCI), and AD dementia enrolled in the Alzheimer’s Disease Neuroimaging Initiative 2 (ADNI2) study. Frailty was operationalized by computing a 40-item Frailty Index (FI). The following AD biomarkers were considered and analyzed according to the participants’ frailty status: CSF Aβ_1-42_, ^181^P-tau, and T-tau; MRI-based hippocampus volume; cortical glucose metabolism at the FDG PET imaging; amyloid deposition at the ^18^F-AV-45 PET imaging. Logistic regression models, adjusted for age, sex, and education, were performed to explore the association of biomarkers with cognitive status at different FI levels. Subjects with higher FI scores had lower CSF levels of Aβ_1-42_, hippocampus volumes at the MRI, and glucose metabolism at the FDG PET imaging, and a higher amyloid deposition at the ^18^F-AV-45 PET. No significant differences were observed among the two frailty groups concerning ApoE genotype, CSF T-tau, and P-tau. Increasing frailty levels were associated with a weakened relationship between dementia and ^18^F-AV-45 uptake and hippocampus volume and with a stronger relationship of dementia with FDG PET. Frailty contributes to the discrepancies between AD pathology and clinical manifestations and influences the association of AD pathological modifications with cognitive changes. AD and dementia should increasingly be conceived as “complex diseases of aging,” determined by multiple, simultaneous, and interacting pathophysiological processes.

## Introduction

The relationship between the biological modifications underlying Alzheimer’s disease (AD) and their phenotypic expression is highly complex. A non-negligible proportion of people diagnosed with AD has few neuropathological abnormalities at autopsy, whereas many cognitively intact individuals exhibit a high burden of AD pathology [1]. Accordingly, a relevant discordance between biomarker- and clinical-based definitions of AD has repeatedly been documented [2]. The understanding of this relationship may be improved by the adoption of constructs and models that comprehensively reflect the biological complexity of the organism as well as the heterogeneity of health trajectories and outcomes within aging. In this regard, the concept of frailty may open promising scenarios in the field.

Frailty is intended as a condition characterized by reduced homeostatic reserves and increased vulnerability to stressors exposing the individual to adverse outcomes [3, 4]. This construct has triggered growing attention in many medical areas [5] as it likely contributes to the relevant variability of health outcomes. Furthermore, it may affect the health trajectories of individuals presenting similar risk profiles (e.g., diagnosed with the same disease) [6].

Frailty is frequently operationalized using a deficit accumulation approach [7]. According to this model, the individual’s degree of frailty is related to the extent of the health deficits he/she has accumulated during the life course. The one’s biological complexity and risk profile can therefore be estimated by quantifying (i.e., arithmetically counting) these negative attributes and condensing them in a single continuous variable, the so-called Frailty Index (FI) [6].

Frailty as accumulation of deficits has already been investigated in the field of dementia and cognitive disorders. It has been shown to independently predict incident dementia among cognitively normal older individuals [8, 9]. Increasing FI scores have been associated with a higher probability of conversion to dementia in subjects with mild cognitive impairment (MCI), and poorer outcomes (i.e., mortality, hospitalization, and steeper worsening of cognitive functioning) in patients with AD [10–12]. Moreover, the FI has been adopted to ascertain the external validity of research protocols enrolling participants with dementia [13]. There is also emerging evidence that frailty, quantified as FI, can influence the neuropathological and biological changes occurring with brain aging and neurodegeneration and the relationship with their phenotypic manifestations [14, 15]. In particular, in a recent analysis of the clinicopathological data from a large sample of community-dwelling older adults, frailty was found to moderate the association between neuropathology and dementia in AD [15].

Based on these premises, it can be hypothesized that the individual’s frailty status may modify the association between candidate AD biomarkers, reflecting in vivo the main neuropathological modifications of the disease, and the cognitive manifestations occurring along the AD continuum [16]. Testing this hypothesis could have important implications since the use of biomarkers is becoming crucial for diagnostic purposes as well as for the identification and development of potential therapeutic targets [17–19]. The present cross-sectional study is aimed at investigating if, and eventually how, frailty influences the changes of the main biomarkers of AD pathology and moderates their relationship with cognitive changes and dementia.

## Methods

### Data sources

Data used in the preparation of this study were obtained from the Alzheimer’s Disease Neuroimaging Initiative (ADNI) database (http://adni.loni.usc.edu). The ADNI was launched in 2003 as a public-private partnership, led by Principal Investigator Michael W. Weiner, MD. The primary goal of ADNI has been to test whether serial magnetic resonance imaging (MRI), positron emission tomography (PET), other biological markers, and clinical and neuropsychological assessment can be combined to measure the progression of MCI and early AD (for up-to-date information, see http://adni.loni.usc.edu).

### Participants and procedures

Data from 778 eligible participants in the ADNI2 study (phase 2 of the ADNI project) were considered for the present analysis. Subjects enrolled in the study were categorized into four diagnostic categories according to their cognitive and functional status:

(i) cognitively normal (CN);
(ii) early MCI (EMCI);
(iii) late MCI (LMCI);
(iv) mild AD dementia.

The diagnosis of MCI was based on the Petersen criteria [20, 21] and required Mini-Mental State Examination (MMSE) scores between 24 and 30 (inclusive), a subjective memory concern, abnormal memory function documented by education-adjusted scores on the Logical Memory II subscale from the Wechsler Memory Scale (WMS)–Revised [22], a clinical dementia rating of 0.5, absence of significant impairment in other cognitive domains, globally preserved activities of daily living, and absence of dementia. All enrolled MCI subjects were, thus, amnestic MCI. For the present purposes, participants with EMCI and LMCI, differing for the degree of memory impairment at the WMS, were grouped together in the MCI category. Patients with AD dementia met the National Institute of Neurological and Communicative Disorders and Stroke and the Alzheimer’s Disease and Related Disorders Association criteria for probable AD [23]. The detailed eligibility and diagnostic criteria can be found in the ADNI2 protocol (http://adni.loni.usc.edu/wp-content/uploads/2008/07/adni2-procedures-manual.pdf).

For each of the considered subjects, data concerning the following domains were downloaded from the ADNI database: demographics; past medical history and comorbidities; general and neurological examination; global cognitive performance, as measured by the MMSE; functional independence, by means of the Functional Assessment Questionnaire (FAQ); apolipoprotein E (ApoE) genotype; cerebrospinal fluid (CSF) concentrations of amyloid beta (Aβ_1-42_,), ^181^phospho-tau (P-tau), and total tau (T-tau); MRI-based measurement of hippocampus volumes; cortical glucose metabolism at the fluorodeoxyglucose (FDG) PET imaging; amyloid deposition as measured by the uptake of ^18^F-AV-45 (i.e., florbetapir) at the PET imaging. All this information referred to the assessments performed during the screening and baseline visits, separated by a maximum window of 28 days.

The detailed description of the different diagnostic procedures, protocols, and measurements is available in the ADNI manual (http://adni.loni.usc.edu) and previous publications [24–27]. The choice of the biomarkers of interest was based on the currently proposed frameworks for the biological definition of AD [18].

### Frailty Index

A FI was operationalized from health variables collected at the screening and baseline visits, following a standard procedure [28]. Candidate variables were incorporated in the FI if they individually met the following criteria:

(i) they must represent health-related deficits, such as symptoms, signs, diseases, and functional impairments, all associated with negative outcomes;
(ii) their prevalence must generally increase with advancing chronological age;
(iii) they must not saturate too early or too late (i.e., they should not be present in > 80% or in < 1% of the study population, respectively);
(iv) they must contain < 5% missing values.

Moreover, the identified set of deficits must also respond to some overall requirements such as covering multiple organ systems and grouping at least 30 variables. Based on the study aims, variables strongly related to dementia and cognitive status were not considered in the computation. The 40 deficits included in the present FI are listed in Table 1. Each variable was coded as 0 (i.e., absent or normal) or 1 (i.e., present or abnormal). The ability to perform daily activities, as measured by the FAQ, was instead more finely graded (i.e., “normal” = 0; “has difficulty but does by self” = 0.25; “requires assistance” = 0.5; “dependent” = 1).

**Table 1:** Deficits considered in the computation of the Frailty Index

Items	Scoring			
	0	0.25	0.5	1
1. Renal-genitourinary diseases	No	-	-	Yes
2. Dermatologic-connective diseases	No	-	-	Yes
3. Hepatic diseases	No	-	-	Yes
4. Cardiovascular diseases	No	-	-	Yes
5. Endocrine-metabolic diseases	No	-	-	Yes
6. Neurological (non-AD) diseases	No	-	-	Yes
7. Psychiatric diseases	No	-	-	Yes
8. Malignancies	No	-	-	Yes
9. Musculoskeletal diseases	No	-	-	Yes
10. Gastrointestinal diseases	No	-	-	Yes
11. Respiratory diseases	No	-	-	Yes
12. Head, eyes, ears, nose, and throat diseases	No	-	-	Yes
13. Hematopoietic-lymphatic diseases	No	-	-	Yes
14. Urinary discomfort	Absent	-	-	Present
15. Shortness of breath	Absent	-	-	Present
16. Low energy	Absent	-	-	Present
17. Falls	Absent	-	-	Present
18. Insomnia	Absent	-	-	Present
19. Constipation	Absent	-	-	Present
20. Drowsiness	Absent	-	-	Present
21. Dizziness	Absent	-	-	Present
22. Musculoskeletal pain	Absent	-	-	Present
23. Seated BP diastolic	≤ 90 mmHg	-	-	> 90 mmHg
24. Seated BP systolic	≤ 140 mmHg	-	-	> 140 mmHg
25. Tremor	Absent	-	-	Present
26. Motor strength	Normal	-	-	Abnormal
27. Gait	Normal	-	-	Abnormal
28. Cerebellar - finger to nose	Normal	-	-	Abnormal
29. Agitation/aggression (NPI)	No	-	-	Yes
30. Anxiety (NPI)	No	-	-	Yes
31. Apathy/indifference (NPI)	No	-	-	Yes
32. Irritability/lability (NPI)	No	-	-	Yes
33. Heating water, making a cup of coffee (FAQ)	0	1	2	3
34. Traveling out of the neighborhood (FAQ)	0	1	2	3
35. Preparing a balanced meal (FAQ)	0	1	2	3
36. Writing checks, paying bills, or balancing checkbook (FAQ)	0	1	2	3
37. Paying attention to and understanding a TV program, book, or magazine (FAQ)	0	1	2	3
38. Playing a game of skill such as bridge or chess (FAQ)	0	1	2	3
39. Shopping alone for clothes, household (FAQ)	0	1	2	3
40. Assembling tax records, business affairs (FAQ)	0	1	2	3

*FAQ* Functional Assessment Questionnaire, *NPI* Neuropsychiatric Inventory

The FI was calculated by dividing the sum of deficits presented by the individual divided for the number of considered deficits (i.e., 40). For instance, a participant presenting 10 out of the 40 deficits had a resulting FI of 0.25 (i.e., 10/40).

The variables needed to compute the 40-item FI were available for 778 out of the 789 participants to the ADNI2 protocol.

### Statistical analysis

Descriptive analyses were conducted to present the characteristics of the study sample. Participants were categorized into two groups according to their frailty status, using the median value of the FI as the cut-point. Unpaired two-sided heteroscedastic *T*-tests or one-way ANOVA (for continuous variables) and two-sided chi-square tests (for categorical variables) were performed to compare the characteristics of participants and biomarkers by frailty and cognitive status. Spearman’s correlation coefficients were calculated to assess the strength and direction of the correlation between FI and age. For each of the considered biomarkers, two levels (i.e., low or high) were created adopting the median as the cut-point.

Biomarkers resulting as significantly different between the two frailty groups at the univariate analysis were then included as binary independent variables in logistic regression models exploring their association with cognitive status (i.e., AD or MCI vs. CN) (binary dependent variable) at the two levels of FI. Interaction (on the multiplicative scale) between FI and each biomarker in their association with cognitive status was calculated as the ratio between the odds ratio (OR) of association between biomarkers and cognitive status in the high FI group and the OR of association in the low FI group. That is, OR_interaction_ = OR_highFI_/OR_lowFI_ [29]. All models were adjusted for age (continuous variable), sex (binary variable), and education (continuous variable). Sensitivity analyses were conducted using: (i) a modified 32-item FI not incorporating functional deficits (i.e., items 33 to 40 of the original FI) to avoid possible overlaps with diagnostic classifications (i.e., MCI and dementia); and (ii) different cutoffs that had been previously adopted in the ADNI study to classify the ^18^F-AV-45, CSF Aβ_1-42_, FDG, and hippocampus volume status [30–32]. ApoE status was not included as a confounder in the models because not significantly different in the two frailty groups at the univariate analysis.

The accuracy of the median FI score at detecting dementia was estimated by the area under the ROC curve (AUC). Specifically, the sensitivity, specificity, and AUC for the median FI cut-point were calculated .

The level of statistical significance was set at *p* < 0.05. All analyses were performed using SPSS version 25 for Mac.

## Results

Overall, 291 CN subjects, 338 participants with MCI, and 149 patients with mild AD dementia were considered in the present study. Their sociodemographic and clinical characteristics are summarized in Tables 2 and 3.

**Table 2:** Sociodemographic and clinical characteristics of participants according to their cognitive status

	CN (*n* = 291)	MCI (*n* = 338)	AD dementia (*n* = 149)	*p*
Age (years)				
Mean ± SD Median (IQR)	73.0 ± 6.0 72.5 (68.3–77.0)	71.6 ± 7.4 71.9 (66.4–76.8)	74.6 ± 8.2 75.2 (70.4–80.2)	< 0.001
Women, *n* (%)	157 (54.0)	153 (45.3)	62 (41.6)	0.023
Education (years)				
Mean ± SD Median (IQR)	16.6 ± 2.5 16.0 (15.0–18.0)	16.3 ± 0.1 16.0 (14.0–18.0)	15.8 ± 2.7 16.0 (14.0–18.0)	0.009
Frailty Index				
Mean ± SD Median (IQR)	0.18 ± 0.08 0.18 (0.13–0.23)	0.21 ± 0.09 0.20 (0.15–0.28)	0.26 ± 0.09 0.26 (0.20–0.33)	< 0.001
MMSE				
Mean ± SD Median (IQR)	29.0 ± 1.2 29.0 (29.0–30.0)	28.0 ± 1.7 28.0 (27.0–29.0)	23.1 ± 2.1 23.0 (21.0–25.0)	< 0.001
ApoEε4, *n* (%)				< 0.001
1 ε4 allele	81 (27.9)	131 (39.1)	69 (47.6)	
2 ε4 allele	7 (2.4)	38 (11.3)	28 (19.3)	
CSF Aβ_1-42_ (pg/ml)				
Mean ± SD Median (IQR)	1237.0 ± 437.5 1306.0 (867.5–1700.0)	980.7 ± 413.2 876.7 (664.7–1293.0)	691.8 ± 322.0 622.5 (489.4–785.8)	< 0.001
CSF T-tau (pg/ml)				
Mean ± SD Median (IQR)	238.4 ± 92.4 213.9 (174.4–298.2)	280.0 ± 134.0 247.5 (186.5–331.5)	373.1 ± 153.2 331.5 (266.8–445.4)	< 0.001
CSF ^181^P-tau (pg/ml)				
Mean ± SD Median (IQR)	21.8 ± 9.4 19.3 (15.3–26.3)	27.0 ± 14.9 23.2 (16.8–33.0)	36.8 ± 16.0 33.2 (25.2–45.5)	< 0.001
MRI hippocampus (ml)				
Mean ± SD Median (IQR)	7.5 ± 0.9 7.5 (7.0–8.0)	7.0 ± 1.1 7.0 (6.3–7.8)	5.9 ± 9.7 5.8 (5.2–6.6)	< 0.001
FDG PET (metaROI)				
Mean ± SD Median (IQR)	1.32 ± 0.11 1.32 (1.25–1.39)	1.25 ± 0.13 1.25 (1.17–1.33)	1.07 ± 0.15 1.07 (0.98–1.17)	< 0.001
^18^F-AV-45 (SUVR)				
Mean ± SD Median (IQR)	1.12 ± 0.18 1.06 (1.00–1.19)	1.23 ± 0.23 1.19 (1.02–1.40)	1.40 ± 0.22 1.43 (1.27–1.54)	< 0.001

*AD* Alzheimer’s disease, *ApoE* apolipoprotein E, ^*18*^*F-AV-45* florbetapir, *CN* cognitively normal, *CSF* cerebrospinal fluid, *FDG* fluorodeoxyglucose (18F), *MCI* mild cognitive impairment, *MMSE* Mini-Mental State Examination, *MRI* magnetic resonance imaging, *PET* positron emission tomography

Missing data: ApoE genotype: *n* = 8; CSF Aβ_1-42_: *n* = 82; CSF T-tau: *n* = 82; CSF ^181^P-tau: *n* = 82; MRI hippocampus: *n* = 87; FDG PET: *n* = 13; ^18^F-AV-45: *n* = 22. The statistical significance was computed by the two-sided chi-square test for sex and ApoE genotype; by one-way ANOVA otherwise

**Table 3:** Sociodemographic and clinical characteristics of participants according to their frailty status

	Overall (*n* = 778)	FI ≤ 0.20 (*n* = 412)	FI > 0.20 (*n* = 366)	*p*
Age (years)				
Mean ± SD Median (IQR)	72.7 ± 7.2 72.7 (67.7–77.6)	71.6 ± 6.7 71.3 (66.9–76.2)	73.9 ± 7.5 73.9 (68.9–79.1)	< 0.001
Sex, *n* (%)				0.03
Women	372 (47.8)	212 (51.5)	160 (43.7)	
Men	406 (52.2)	200 (48.5)	206 (56.3)	
Education (years)				
Mean ± SD Median (IQR)	16.3 ± 2.6 16.0 (14.0–18.0)	16.6 ± 2.5 16.0 (15.0–18.0)	16.0 ± 2.8 16.0 (14.0–18.0)	< 0.01
MMSE				
Mean ± SD Median (IQR)	27.4 ± 2.7 28.0 (26.0–30.0)	28.1 ± 2.1 29.0 (27.0–30.0)	26.7 ± 3.1 28.0 (25.0–29.0)	< 0.001
Diagnosis, *n* (%)				< 0.001
CN	291 (37.4)	200 (48.5)	91 (24.9)	
MCI	338 (43.4)	172 (41.8)	166 (45.3)	
AD dementia	149 (19.2)	40 (9.7)	109 (29.8)	
ApoE genotype, *n* (%)				0.28
1 ε4 allele	281 (36.5)	143 (34.9)	138 (38.3)	
2 ε4 allele	73 (9.5)	35 (8.5)	38 (10.6)	
CSF Aβ_1-42_ (pg/ml)				
Mean ± SD Median (IQR)	1020.2 ± 451.1 909.5 (652.0–1434.8)	1105.2 ± 447.2 1006.0 (715.5–1620.0)	925.4 ± 437.1 785.8 (586.9–1230.0)	< 0.001
CSF T-tau (pg/ml)				
Mean ± SD Median (IQR)	282.3 ± 133.2 250.4 (189.0–340.4)	275.0 ± 128.3 241.0 (189.7–321.5)	290.4 ± 138.3 263.9 (188.1–359.8)	0.13
CSF ^181^P-tau (pg/ml)				
Mean ± SD Median (IQR)	27.0 ± 14.4 23.4 (17.1–32.8)	26.2 ± 14.1 22.3 (17.1–31.1)	27.8 ± 14.6 25.3 (17.1–34.2)	0.13
MRI hippocampus (ml)				
Mean ± SD Median (IQR)	7.0 ± 1.2 7.1 (6.2–7.8)	7.3 ± 1.2 7.3 (6.6–8.0)	6.7 ± 1.1 6.8 (5.9–7.5)	< 0.001
FDG PET (metaROI)				
Mean ± SD Median (IQR)	1.24 ± 0.16 1.26 (1.15–1.34)	1.27 ± 0.14 1.29 (1.20–1.36)	1.21 ± 0.16 1.22 (1.11–1.33)	< 0.001
^18^F-AV-45 PET (SUVR)				
Mean ± SD Median (IQR)	1.22 ± 0.23 1.15 (1.02–1.40)	1.19 ± 0.22 1.10 (1.02–1.34)	1.25 ± 0.24 1.24 (1.03–1.43)	< 0.001

*AD* Alzheimer’s disease, *ApoE* apolipoprotein E, ^*18*^*F-AV-45* florbetapir, *CN* cognitively normal, *CSF* cerebrospinal fluid, *FDG* fluorodeoxyglucose (18F), *MCI* mild cognitive impairment, *MMSE* Mini-Mental State Examination, *MRI* magnetic resonance imaging, *PET* positron emission tomography

Missing data: ApoE genotype: *n* = 8; CSF Aβ_1-42_: *n* = 82; CSF T-tau: *n* = 82; CSF ^181^P-tau: *n* = 82; MRI hippocampus: *n* = 87; FDG PET: *n* = 13; ^18^F-AV-45: *n* = 22. The statistical significance was computed by the two-sided chi-square test for sex, diagnosis, and ApoE genotype; by the two-sided *T*-test otherwise

**Fig. 1:**
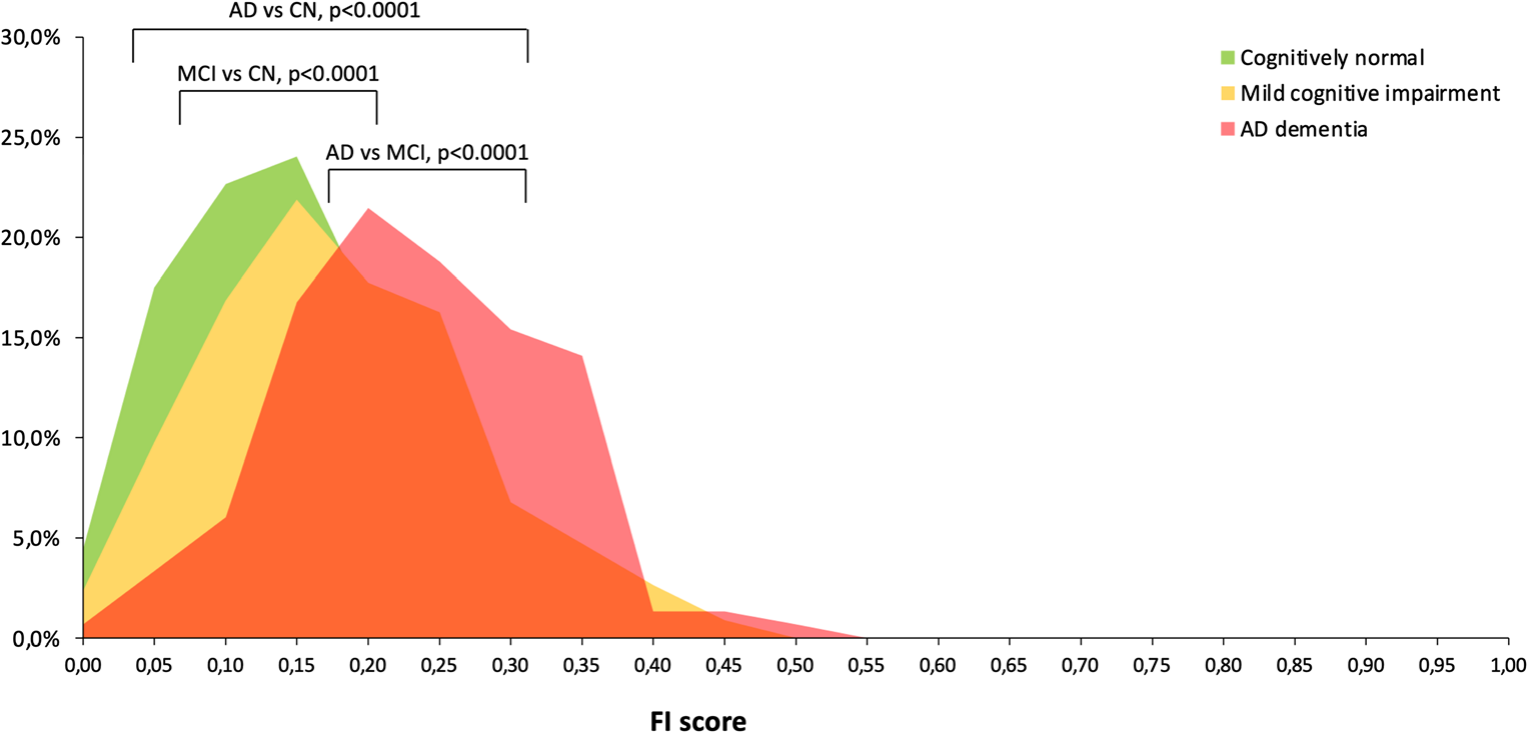
Distribution of the Frailty Index in the three cognitive groups (cognitively normal: green; mild cognitive impairment: yellow; AD dementia: red). The comparison of the three distributions of the Frailty Index is based on the Kolmogorov-Smirnov two-sided test

The FI had a characteristic right-skewed distribution, both in the whole sample and in each of the three diagnostic groups (Fig. 1), and ranged between 0 and 0.56. The median FI score was 0.20 (IQR = 0.14–0.27) and the 99th percentile was 0.44.

The FI exhibited a statistically significant correlation with participants’ chronological age (Spearman’s rho = 0.20; *p* < 0.001). Subjects with higher FI values (i.e., > 0.20; *n* = 366) were significantly older, less educated, more frequently male, more likely to have a diagnosis of dementia, and had lower MMSE scores compared to those with a FI score lower than the median value (Table 3). Moreover, they had lower CSF levels of Aβ_1-42_, hippocampus volumes at the MRI, and glucose metabolism at the FDG PET imaging, and a higher amyloid deposition at the ^18^F-AV-45 PET. Conversely, no significant differences were observed among the two frailty groups concerning ApoE genotype, and CSF levels of T-tau and P-tau (Table 3).

A limited concordance was observed between AD biomarkers and the cognitive status of participants in the overall sample. Indeed, a proportion ranging between 19.8 and 22.8% of those with an abnormal biomarker status (i.e., CSF Aβ_1-42_, hippocampus volume, and FDG uptake values lower than median cut-points, ^18^F-AV-45 uptake higher than the median value) had normal cognitive functioning. On the other hand, a relevant proportion of those with normal biomarkers, varying between 45.0 and 46.6%, met the criteria for MCI or mild AD dementia (Table 4). Among participants with abnormal biomarker values, the prevalence of dementia was higher in those with higher FI scores compared to those with FI ≤ 0.20 (i.e., 42.9% vs. 18.7% for high ^18^F-AV-45 uptake; 43.1% vs. 19.2% for low CSF Aβ_1-42_; 42.9% vs. 17.5% for low hippocampus volume; 46.7% vs. 19.1 for low FDG uptake). Conversely, in the case of normal biomarker status, the proportion of CN cases was higher in those with lower FI scores (i.e., 62.9% vs. 41.7% for low ^18^F-AV-45 uptake; 59.3% vs. 43.9% for high CSF Aβ_1-42_; 61.3% vs. 43.0% for high hippocampus volume; 61.1% vs. 45.1% for high FDG uptake) (Table 4).

**Table 4:** Proportion of participants with normal cognition, MCI, or AD dementia according to Frailty Index values by each of the considered biomarkers. Data are shown as *n* (%)

		Frailty Index								
		Low			High			Total		
		CN	MCI	AD dem.	CN	MCI	AD dem.	CN	MCI	AD dem.
^18^F-AV-45	*Low*	144 (62.9)	78 (34.1)	7 (3.0)	63 (41.7)	70 (46.4)	18 (11.9)	207 (54.5)	148 (38.9)	25 (6.6)
	**High**	49 (28.7)	90 (52.6)	32 (18.7)	27 (13.2)	90 (43.9)	88 (42.9)	76 (20.2)	180 (47.9)	120 (31.9)
CSF Aβ_1-42_	**Low**	47 (31.1)	75 (49.7)	29 (19.2)	22 (11.2)	90 (45.7)	85 (43.1)	69 (19.8)	165 (47.4)	114 (32.8)
	*High*	128 (59.2)	82 (38.0)	6 (2.8)	58 (43.9)	63 (47.8)	11 (8.3)	186 (53.4)	145 (41.7)	17 (4.9)
MRI hippocampus	**Low**	48 (33.6)	70 (48.9)	25 (17.5)	31 (15.3)	85 (41.9)	87 (42.8)	79 (22.8)	155 (44.8)	112 (32.4)
	*High*	133 (61.3)	76 (35.0)	8 (3.7)	55 (43.0)	61 (47.6)	12 (9.4)	188 (54.5)	137 (39.7)	20 (5.8)
FDG PET	**Low**	55 (31.8)	85 (49.1)	33 (19.1)	26 (12.2)	88 (41.1)	100 (46.7)	81 (20.9)	173 (44.7)	133 (34.4)
	*High*	143 (61.1)	84 (35.9)	7 (3.0)	65 (45.1)	74 (51.4)	5 (3.5)	208 (55.0)	158 (41.8)	12 (3.2)

Low: ≤ median value; high: > median value

Italic: normal; bold: abnormal

Median values: ^18^F-AV-45: 1.15 SUVR; CSF Aβ_1-42_: 909.5 pg/ml; MRI hippocampus: 7.1 ml; FDG PET: 1.26 metaROI

Available data: ^18^F-AV-45: *n* = 756; CSF Aβ_1-42_: *n* = 696; MRI hippocampus: *n* = 691; FDG PET: *n* = 765

The relationship between each AD biomarker and cognitive status was influenced by frailty levels (Table 5). In particular, increasing frailty levels were associated with a weaker relationship between dementia and ^18^F-AV-45 uptake (OR_interaction_ = 0.58; 95% CI: 0.37–0.77), and hippocampus volume (OR_interaction_ = 0.86; 95% CI: 0.64–0.95). On the contrary, the association of dementia with FDG PET (OR_interaction_ = 3.86; 95% CI: 2.37–5.77) was stronger at higher FI scores. Finally, no significant interaction was observed with CSF Aβ_1-42_ (OR_interaction_ = 1.30; 95% CI: 0.93–1.52). Similar results were obtained when the modified 32-item FI, not including functional deficits, was used. Indeed, the increase in FI scores weakened the relationship between dementia and amyloid deposition, and hippocampus volume, while strengthening the relationship between dementia and FDG PET (data not shown). The adoption of the cut-points derived from the official ADNI protocol and previous studies (i.e., 1.11 SUVR for ^18^F-AV-45, 880 pg/ml for CSF Aβ_1-42_, 1.21 metaROI for FDG PET, and 6723 ml for hippocampus volume) did not substantially change the results in sensitivity analyses (Table 6).

**Table 5:** Results of logistic regression models exploring the association between dichotomized biomarkers (independent variables of interest) and AD dementia/MCI status (dependent variables of interest) stratified by Frailty Index values

	Frailty Index				Interaction	
	Low		High			
AD dem. (vs. CN reference group)	OR	95% CI	OR	95% CI	OR	95% CI
^18^F-AV-45 (high vs. low)	19.49	7.69–49.41	11.32	5.61–22.83	0.58	0.37–0.77
CSF Aβ_1-42_ (low vs. high)	15.51	5.90–40.79	20.23	8.93–45.82	1.30	0.93–1.52
MRI hippocampus (low vs. high)	19.17	6.98–52.64	16.45	7.26–37.27	0.86	0.64–0.95
FDG PET (low vs. high)	13.46	5.49–32.97	51.90	18.61–144.80	3.86	2.37–5.77
MCI (vs. CN reference group)	OR	95% CI	OR	95% CI	OR	95% CI
^18^F-AV-45 (high vs. low)	4.01	2.51–6.40	3.41	1.92–6.07	0.85	0.37–0.98
CSF Aβ_1-42_ (low vs. high)	2.69	1.68–4.30	3.92	2.13–7.21	1.46	0.53–1.96
MRI hippocampus (low vs. high)	3.69	2.19–6.21	3.92	2.10–7.33	1.06	0.51–1.13
FDG PET (low vs. high)	2.82	1.81–4.39	3.28	1.84–5.86	1.16	0.46–1.34

Each biomarker (categorical high vs. low or low vs. high, independent variable) is singularly included in the age-, sex-, and education-adjusted regression model to predict cognitive status (AD dem. vs. CN or MCI vs. CN, dependent variable). The population is stratified according to low or high Frailty Index values

Low: ≤ median value; high: > median value

Median values: ^18^F-AV-45: 1.15 SUVR; CSF Aβ_1-42_: 909.5 pg/ml; MRI hippocampus: 7.1 ml; FDG PET: 1.26 metaROI

**Table 6:** Results of logistic regression models exploring the association between dichotomized biomarkers (independent variables of interest) and AD dementia status (dependent variables of interest) stratified by Frailty Index values

	Frailty Index				Interaction	
	Low		High			
AD dem. (vs. CN reference group)	OR	95% CI	OR	95% CI	OR	95% CI
^18^F-AV-45 (> 1.11 SUVR)	16.36	6.32–42.33	12.28	5.99–25.21	0.75	0.51–0.90
CSF Aβ_1-42_ (< 880 pg/ml)	8.94	4.13–19.34	11.24	5.70–22.17	1.26	0.79–1.49
MRI hippocampus (< 6.7 ml)	15.54	6.66–36.23	11.57	5.70–23.48	0.74	0.50–0.90
FDG PET (< 1.21 metaROI)	17.67	7.69–40.63	26.63	12.29–57.70	1.51	1.08–1.85

Each biomarker (categorical, independent variable) is singularly included in the age-, sex-, and education-adjusted regression model to predict cognitive status (AD dem. vs. CN, dependent variable) at low and high Frailty Index values

The cut-points for each of the considered biomarkers were derived from [30–32]

As to MCI subjects, only the relationship with ^18^F-AV-45 was significantly influenced by FI scores (OR_interaction_ = 0.85; 95% CI: 0.37–0.98) (Table 5).

ROC curve analysis (AUC 0.71; 95% CI 0.67–0.75, *p* < 0.001) showed that the adopted FI cut-point (i.e., 0.20) had an accuracy of nearly 70% and a sensitivity of 73.2% in discriminating participants with and without mild AD dementia.

## Discussion

To the best of our knowledge, this study constitutes the first attempt to test the hypothesis that frailty may act as a latent factor in the relationship between AD candidate biomarkers and the phenotypic expression of the neurodegenerative condition. The present results confirm and extend the previous findings obtained by Wallace and colleagues based on clinicopathological data [15], that is:

(i) frailty contributes to the discrepancies between AD pathology and clinical manifestations; and
(ii) frailty influences the association of AD pathological modifications with the individual’s cognitive changes (i.e., MCI and dementia).

In the present analysis, approximately one out of five participants had a positive AD biomarker without presenting any cognitive disturbance and slightly less than one out of two met the MCI or AD dementia criteria in the absence of any biomarkers’ change. The rate of misclassification was significantly influenced by frailty levels. The probability of being cognitively intact in the presence of a positive biomarker was higher among subjects with lower FI scores (i.e., 28.7–33.6% vs. 11.2–15.3%). Conversely, participants with higher FI values had an increased likelihood of presenting MCI or dementia despite the normality of biomarkers (cumulative 54.9–58.3% vs. 37.1–40.7%). Two main reflections are inspired by these findings and are aligned with the interpretations proposed by Wallace et al. [15]. First, individuals with a lower amount of health deficits seem better able to cope with the accumulation of AD neuropathology. Consistent with its definition, frailty emerges as a reduction of those reserves which enable the organism (and the brain) to tolerate the onset of pathological perturbations and modifications with limited functional consequences. The progressive accumulation of deficits lowers the threshold for AD pathological changes to produce cognitive deficits. Second, frailty likely concurs to the decline of cognitive functioning and the development of dementia through pathophysiological mechanisms that are not directly captured by candidate AD biomarkers. Frailty has already been shown to be directly associated with the main modifications of AD, such as amyloid deposition and brain atrophy [33]. Nevertheless, it is accompanied by additional biological processes (e.g., inflammation, immunosenescence, metabolic and energetic declines, loss of proteostasis) that may synergistically contribute to the onset of dementia [34].

It is noteworthy that the individual relationships between each of the considered biomarkers and cognitive status were differently moderated by frailty. Indeed, the increase in FI weakened the association of ^18^F-AV-45 PET status and MRI hippocampal volume with AD dementia. On the other hand, it strengthened the relationship between FDG PET status and AD dementia. In other words, the likelihood that a positive amyloid PET scan and an MRI-based evidence of hippocampus atrophy manifested with AD dementia was higher among participants with lower frailty levels. Conversely, higher frailty scores markedly increased the probability that brain hypometabolism resulted in an overt dementia condition. These findings suggest that the pathogenic contribution of some of the AD pathophysiological processes (i.e., amyloid deposition, neuronal loss) is significantly influenced by the background noise of the organism’s biology [35]. Therefore, the interpretation of the changes affecting the relative biomarkers may be biased by the individual’s biological complexity. Consequently, the current research approach (and the proposed diagnostic framework [18]) based on a bivariate conception of the biomarker is probably inadequate and results in a reductionistic picture of a complex phenomenon. The introduction of a third dimension, such as frailty, may instead enhance the understanding of the multifaceted pathways leading to neurodegeneration, their link with phenotypic manifestations and clinical diagnoses, and possibly their suitability as research targets. In this regard, it should be observed that tau deposition was not affected by frailty as suggested by the detection of similar CSF levels of T-tau and ^181^P-tau in the two FI groups (Table 1). Thus, the cascade of molecular events leading to the formation of neurofibrillary tangles may theoretically be regarded as more specific of neurodegeneration, including AD, compared to other pathways and abnormalities that emerged as likely more consistently shared with the multisystemic aging process. This latter result is in line with the findings by Wallace and colleagues who observed that the interaction between frailty and dementia status was essentially driven by amyloid rather than tau pathology [15]. Interestingly, the findings on the interaction between frailty and amyloid deposition were not fully concordant when it was measured in terms of florbetapir uptake (significant interaction with frailty) or CSF Aβ_1-42_ levels (non-significant interaction with frailty) (Table 5). However, it is noteworthy that a modest agreement between the two biomarkers was observed in the sample (kappa = 0.66; *p* < 0.001), with nearly 17% of subjects being discordantly classified based on the two diagnostic procedures.

Overall, the present findings may have important practical implications. The routine adoption of a versatile measure such as the FI may provide useful, additional information when exploring the clinical expression of AD and assessing the presence of cognitive disorders in older individuals. This may also be the case in hyper-selected populations of participants in research protocols where monodimensional assessments may fail to adequately discriminate between different clinical and biological profiles. Accordingly, the use of the FI may consent to render more homogeneous the study samples in terms of clinical and biological complexity, thus increasing the external validity of the observed findings [13]. Also, this tool may support a person-centered reading of the results from biomarker assessments, thus attributing the proper weight to the biological abnormalities documented in a given individual. This may ultimately sustain personalized therapeutic approaches.

This study has several limitations to be mentioned. The cross-sectional design impedes to infer about causality in the observed relationship between frailty, AD pathology, and cognitive status. Longitudinal data on the modifications of the factors are fundamental to better elucidate their interaction. The analysis was conducted in a highly selected sample of subjects that are probably not representative of the “real world” population of older people with intact or declining cognition [36]. Individuals with unstable or severe medical conditions (e.g., stroke, cancer, health failure) and MRI evidence of relevant brain vascular pathology (i.e., infarctions, multiple and/or strategic lacunes) were, in fact, not considered for participation in the ADNI study. This has probably attenuated the overall frailty levels of the considered subjects and limited the possibility of properly accounting for the effect of additional pathophysiological mechanisms (e.g., vascular pathology) that have been frequently associated with dementia and commonly contribute to mixed neuropathologies [37]. The decision to base the present analyses on the median values of the considered biomarkers rather than on clinically validated cut-points has potentially contributed to misclassify a non-negligible share of participants. Nevertheless, this approach was motivated by the fact that, in the ADNI study, multiple cutoffs have been calculated for each biomarker thus making it arbitrary the choice on which of them to rely on. Anyway, the use of ADNI-derived cutoff values in sensitivity analyses did not substantially change the findings.

`In conclusion, the present study confirms that frailty influences the neuropathophysiology and clinical expression of AD. The age-related accumulation of health deficits significantly moderates the association between several pathways implicated in the pathogenesis of the disease and its phenotypic manifestations. Moreover, it affects the individual threshold at which neuropathological changes result in clinical impairments and diagnostic entities. AD and dementia should increasingly be conceived as “complex diseases of aging” [15], determined by multiple, simultaneous, and interacting pathophysiological processes, thus abandoning one-fits-all and reductionist interpretations. In this alternative framework, the adoption of the frailty construct may improve our comprehension of the biological modifications contributing to dementia, consent to better interpret the findings of diagnostic procedures, and possibly better calibrate therapeutic targets and interventions.

## Data Availability

Data used in preparation of this article were obtained from the Alzheimer’s Disease Neuroimaging Initiative (ADNI) database (adni.loni.usc.edu).
